# “WHAT STANDARD SHOULD WE SET?”: A QUALITATIVE STUDY OF REHABILITATION PROFESSIONALS’ PERSPECTIVES ON REHABILITATION NEEDS AFTER TRAUMATIC INJURIES

**DOI:** 10.2340/jrm.v57.44087

**Published:** 2025-10-30

**Authors:** Emilie ISAGER HOWE, Helene L. SØBERG, Cecilie RØE, Marianne LØVSTAD, Nada ANDELIC

**Affiliations:** 1 Department of Physical Medicine and Rehabilitation, Oslo University Hospital, Oslo; 2 Centre for Habilitation and Rehabilitation Models and Services (CHARM), Institute of Health and Society, University of Oslo, Oslo; 3 Faculty of Health Sciences, Oslo Metropolitan University, Oslo; 4 Institute of Clinical Medicine, Faculty of Medicine, University of Oslo, Oslo; 5 Department of Research, Sunnaas Rehabilitation Hospital, Bjørnemyr; 6 Department of Psychology, Faculty of Social Sciences, University of Oslo, Oslo, Norway

**Keywords:** physical trauma, focus groups, rehabilitation, healthcare professionals

## Abstract

**Objective:**

To assess rehabilitation professionals’ perspectives on unmet rehabilitation needs in patients with traumatic injuries and how to bridge the gap between met and unmet needs.

**Design:**

Semi-structured focus-group interviews analysed using a reflexive thematic approach.

**Methods:**

Eighteen strategically sampled health professionals (67% female, aged 27–56 years) from specialist and community-based rehabilitation services in Northern and South-Eastern Norway were interviewed regarding their perspectives on alignment of current rehabilitation services with patient needs, reasons for misalignment, and potential service improvements.

**Results:**

Care continuity and multidisciplinary collaboration was identified as essential for high-quality rehabilitation services. Unmet needs were noted in community-based services for cognitive and mental health support, as well as financial assistance for patients. Health professionals faced challenges prioritizing patient needs and expressed frustration with not being able to provide legally mandated services. Contributing factors included insufficient resources, lack of competency standards, and weak organizational structure. Suggested improvements were early rehabilitation assessments and stronger collaboration between specialist and community services.

**Conclusions:**

Strengthening community-based rehabilitation for traumatic injuries requires establishing professional competency standards, stronger coordination across care levels, and greater integration of psychosocial support. Advancing these dimensions calls for critical reflection on clinical practice, service organization, and health policy.

## LAY ABSTRACT

The aim of this study was to explore healthcare professionals’ perspectives on the rehabilitation needs of individuals with physical traumatic injuries. Rehabilitation professionals were interviewed regarding what rehabilitation services they thought the patients needed and whether current services are in line with those needs. They also reflected on reasons why services may not be in line with rehabilitation needs and ways these services can be improved. The participants specifically highlighted a need to improve rehabilitation services to individuals with traumatic injuries after they are discharged from hospital. Limited resources, lack of training and competency standards, and poor service organization were among the listed reasons for unmet rehabilitation needs. Suggestions for improvement included early assessment of needs, facilitating and strengthening the collaboration between specialized and community-based services, in addition to increased focus on rehabilitation within programmes training future rehabilitation professionals.

Traumatic injury, defined as a physical injury with sudden onset and potentially fatal or non-fatal consequences, is a leading cause of mortality and functional disability in children and young adults (1). Traumatic injuries can result in impairments across multiple domains, requiring both short- and long-term rehabilitation. Studies indicate that early initiation of intensive rehabilitation improves patient outcomes and is cost-effective (2–4). However, unmet rehabilitation needs remain common (5–7).

A scoping review on need for rehabilitation services identified a substantial gap between demand and available services across countries (8). The lack of strategic planning for both specialized and community-based rehabilitation has resulted in uneven service distribution, limited capacity, and inadequate infrastructure, creating major barriers to post-trauma care. A recent mixed-method systematic review of rehabilitation outcomes by service provision and geographical location further highlighted wide variations in patient experiences, along with limited confidence among some clinicians and local hospitals (9). The authors concluded that future multicentre research is needed to better capture unmet needs, geographical disparities, and clinician perspectives, thereby strengthening capacity and communication across healthcare levels.

A cohort study found unmet needs for community-based rehabilitation in approximately 50% of individuals 6–12 months after moderate-to-severe traumatic injury (10). Unmet healthcare and rehabilitation needs persist in up to 65% of individuals several years after traumatic brain injury (TBI) and spinal cord injury (SCI) (7, 11). Psychological services and cognitive rehabilitation were the least provided services relative to documented impairments 3 and 6 months post-TBI (6). Qualitative data suggest that inadequate information, service availability, poorly managed care transitions, and challenges relating to affordability and transportation hinder access to rehabilitation services post-injury (12–16).

International frameworks, such as the World Health Organization Rehabilitation Competency Framework (WHO-RCF) (17) and guidelines for rehabilitation after traumatic injury from the National Institute for Health and Care Excellence (NICE) (18), emphasize early assessment, a person-centred and multidisciplinary approach, continuity of care, and integration of physical, cognitive, and psychosocial support. While these standards outline best practice, perceptions of rehabilitation needs vary among stakeholders and implementation remains inconsistent, resulting in significant variation in access and quality of post-trauma rehabilitation.

This study employs Bradshaw’s taxonomy of need (19) as a theoretical framework to explore healthcare professionals’ perspectives on rehabilitation needs after traumatic injury. Bradshaw’s taxonomy distinguishes between 4 types of needs: *normative* (defined by professional standards or expert opinion), *felt* (service users’ perceptions), *expressed* (felt need translated into demand), and *comparative* (discrepancies in service provision among similar groups). Using the concept of normative need, this study aimed to explore rehabilitation professionals’ perspectives on:

- Normative rehabilitation needs of individuals with traumatic injuries. In this context, a normative rehabilitation need was defined as the need for rehabilitation based on professional judgement or rehabilitation guidelines.
- Whether existing rehabilitation services align with normative needs of individuals with traumatic injury.
- Reasons for potential misalignments between provided services and needs.
- Strategies to address discrepancies between rehabilitation needs and services.

Studies performed in the Norwegian context may be of international interest because of a well-organized public trauma system and a publicly funded welfare and healthcare system, including rehabilitation services.

## METHODS

### Study design and context

This qualitative analysis is part of a broader study on user experiences and perspectives on rehabilitation services after traumatic injuries, conducted at Oslo University Hospital. The Regional Committees for Medical and Health Research Ethics in South-Eastern Norway approved all procedures (#372996).

### Participants and procedures

Health professionals from specialist or municipal rehabilitation services were strategically sampled through service coordinators. The inclusion criteria required a minimum of 2 years’ work experience in rehabilitation services, Norwegian residency, and proficiency in Norwegian to participate in focus-group interviews. Participants represented both urban and rural areas in the Northern and South-Eastern health regions of Norway to capture diverse perspectives influenced by structural, organizational, and geographic variations. Participants received information on the project and provided written informed consent. None of the rehabilitation professionals who were approached declined to participate.

Four focus-group interviews, involving a total of 18 participants, were conducted between December 2023 and April 2024. Detailed participant characteristics are presented in Table I. The interviews were performed face-to-face at the participants’ workplaces, lasting 75 to 90 min each. The lead author (EIH), a clinical neuropsychologist, facilitated all interviews. Semi-structured interviews, guided by an interview schedule, explored participant perspectives on current rehabilitation practices, alignment of services with perceived patient needs, reasons for potential misalignments, and suggestions for service improvement. Interviews were audio-recorded and transcribed verbatim by the lead author shortly after completion.

**Table I:** Participant characteristics (*n*=18)

Age, years, mean (range)	41 (27–56)
Sex, female, *n* (%)	12 (67)
Education, years, mean (range)	16 (15–18)
Occupation, *n* (%)	
Physical therapist	6 (33)
Nurse	5 (28)
MD	2 (11)
Occupational therapist	2 (11)
Neuropsychologist	1 (6)
Unit leader/Team coordinator	2 (11)
Experience with rehabilitation services, years, mean (range)	14 (3.5–35)
Setting, *n* (%)	
Specialist rehabilitation services	11 (61)
Community-based rehabilitation services	7 (39)

### Data analysis

The transcripts were coded using NVivo 14 (https://lumivero.com/products/nvivo/). A 6-step reflexive thematic analysis approach guided the analysis (20, 21). Initially, the lead author familiarized herself with the data by reviewing recordings, notes, and transcripts. Systematic initial coding was applied across the dataset, which was then organized into broader preliminary themes. These were further reviewed and refined through discussions between the first and last authors. During the final stage, EIH and NA held regular discussions to reach consensus, identifying 8 overarching themes with associated sub-themes. To incorporate diverse clinical perspectives, all co-authors with expertise in clinical neuropsychology and rehabilitation medicine contributed to the final theme refinement. The findings mark the culmination of the reflexive thematic analysis, aiming to produce a comprehensive report that describes the themes in detail, supported by empirical material such as quotations and in-depth descriptions.

## RESULTS

The analysis yielded several themes within each overarching topic. Themes, sub-themes, and representative quotes are presented in Tables II–V. The themes and sub-themes are presented comprehensively below.

**Table II:** Normative rehabilitation needs as expressed in the focus-group interviews by the 18 participating rehabilitation professionals

Topic 1: The normative rehabilitation needs of individuals with traumatic injuries		
Themes	Sub-themes	Representative quotes
Transdisciplinary work	Multidisciplinary team work	“ … everyone provides their input, perceptions and thoughts regarding what we need to focus most on … we do that regularly, because those meetings are really patient-centred and important for what we do next. Otherwise, it’s just like everyone’s doing their thing, and there’s no real collaboration”
	Goal-setting	“We work very goal-oriented. They [the patient] set very specific activity goals that we work on…. One goal can involve many sub-goals”
Continuity	Predictability	“ … that they know that someone will be there for them when they get back home. That feeling of security is important for us to promote… Always trying to think one step ahead about what type of services they will need”
	Transitions	“It is demanding to manage transitions, it requires resources to follow them up… It is so important in terms of what happens down the road, also for rehabilitation and preventing re-admissions”
	Information flow	“ … to be able to provide the best service to the patient as fast as possible, it is important that we receive good quality information as early as possible. So that we are able to plan ahead”

**Table III:** Alignment of current rehabilitation services with patient needs as expressed in the focus-group interviews by the 18 participating rehabilitation professionals

Topic 2: Whether current rehabilitation services are in line with the normative needs of individuals with traumatic injuries		
Themes	Sub-themes	Representative quotes
Normative needs	Lack of personnel	“We know that the law requires us to have such and such services, but we don’t have enough personnel. There are councils that don’t have a single physical therapist…. So no matter how well-meaning we are, sometimes we hit the wall”
	Financial support	“Maybe 90 per cent of the users that I’ve met working in different districts, have financial difficulties and problems that belong with a social worker…. There are an incredible number of users that don’t have a place to live, who live with their children, and that’s unfortunate…. It’s frustrating spending so much time and resources on it, when you should be focusing on other things. I really feel like that’s a mistake. There’s no money to save by not having a social worker, because it’s something that needs to get done regardless”
	Emotional support	“It’s very difficult to help someone get treatment, because they never really fit the category. What they [the public mental health service] judge as too mild or too severe. I mean, either it’s a natural reaction to losing something in life, or that you’ve lost functions. It’s a natural reaction that doesn’t need treatment. Or it’s too severe psychiatric issues, and then they can’t treat it”
	Cognitive rehabilitation	“ … you can essentially write that the patient has severe cognitive impairment, and that it’s going to last, it’s not going to change after discharge, and he’s going to need follow-up for it. And then there’s no one there at the other end…. Maybe an occupational therapist that disappears after a few weeks”
	Meaningful lives	“Not long ago, we had a young boy who injured himself seriously and will be wheelchair-bound for the rest of his life, and we had a meeting with the school, community-based services, and all the services who are going to help him, and when we finished the meeting, he said ‘you’re just sitting there talking about the help I need to get out of bed, eat, get from A to B, but you say nothing about how I am going to live my life’. And I find that really interesting, because we focus a lot on providing help with morning care, showering, getting into the car, getting into the wheelchair, but then? You’re just supposed to sit there? There’s a missing link”
	Prioritizing	“Not everything is realistic, you know. What level of standard should you set? I’ve just accepted that’s the way it is. It’s a lot meeting all needs, but that you’re able to attend to the most important ones that allow the user to function. It’s not possible to help everyone”

**Table IV:** Reasons for unmet rehabilitation needs as expressed in the focus-group interviews by the 18 participating rehabilitation professionals

Topic 3: Reasons why provided rehabilitation services might not be consistent with the needs of individuals with traumatic injuries		
Themes	Sub-themes	Representative quotes
Resources	Economic	“Rehabilitation isn’t that one hour with a physical or occupational therapist. It is everything that happens in all situations. It’s building competency in the communities so the patients don’t lose their entire network, their job when they are discharged. That will cost. And the funds that are available today aren’t enough”
	Time constraints	“It’s a very short amount of time we’re able to be there…. We’re not able to offer rehabilitation in the longer term. It’s just to maintain things”
Knowledge/skills	Professional competency standards	“That’s one of the challenges, that there’s no established standard for level of competency in the communities”
	Experience	“One thing is the willingness to address it, another is the skill to handle it, to recognize [cognitive impairment]…. Because cognitive rehabilitation is really complex. You have to work with it for a while to recognize those difficulties in the patients”
Service organization	Service availability	“ … huge differences in what’s available, and difficult to find out what the communities can offer. It’s not like you can find everything in one place”
	Allocation of services	“ … these central purchaser offices, the side effect is that they have more experience with assigning services, but the professional expertise isn’t there, and they don’t work with the patient either … the information stops at that level”
	Geographic variations	“It depends on where they [the patient] end up. There are 15 different districts, and they all operate differently”

**Table V:** How to bridge the gap between met and unmet rehabilitation needs as expressed in the focus-group interviews by the 18 participating rehabilitation professionals

Topic 4: Perspectives on how the gap between met and unmet rehabilitation needs can be addressed		
Themes	Sub-themes	Representative quotes
Identifying rehabilitation needs	Early assessment	“ … a more complete evaluation of all patients who may need rehabilitation and maybe initiating rehabilitation earlier, and not least, more long-term follow-up in the specialist healthcare also”
	Intermediary assessment	“ … we need more rehabilitation centres that we can operate ourselves … where you have [patients] who are discharged from hospital … but they can’t go back home, what do we do with them? … where we can assess their functional status and ability to live independently before they go back home”
	Long-term follow up	“ … in an ideal world, a multidisciplinary outpatient clinic that could provide follow-up for a longer period of time”
	Collaborative arenas	“I think it’s very difficult for the community-based health services to make substantial changes. Because, in a way, it blows up the entire municipality structure as it is today…. We have to find other arenas for collaboration … a lot of it has to do with structure”
Perception of rehabilitation	Shared understanding	“I would like to know what people mean by community-based rehabilitation. What is it? A shared understanding of it. That’s what we need. We need to understand that rehabilitation doesn’t equal training. I mean, that rehabilitation is more than just training. It’s returning to a functional life”
	Rehabilitation-specific education	“ … there should be a greater focus on rehabilitation within the bachelor’s degrees…. That would also do something with the culture when you start working. That you’re not just placed there to warm a meal in 10 minutes or spend 20 minutes providing morning care, but that it’s actually rehabilitation in a life-course perspective”

### Topic 1: Normative rehabilitation needs of individuals with traumatic injuries

*Transdisciplinary work*. Participants from both specialized and community-based rehabilitation services emphasized the importance of multidisciplinary teamwork in providing rehabilitation. Key advantages included shared responsibility and understanding of the patient’s recovery process, the integration of diverse perspectives, and the pursuit of common goals. Setting treatment goals collaboratively with patients and systematically working towards them was considered essential to the rehabilitation process.

*Continuity*. Continuous care was deemed crucial for effective rehabilitation services. Participants highlighted various aspects of continuity, such as long-term involvement of care personnel, effective information and knowledge transfer among health professionals across different care levels, and seamless transitions from specialist to community-based rehabilitation services. Rehabilitation professionals emphasized the importance of a predictable process to foster trust and security in patients and their families. Early engagement with patients in the rehabilitation process and anticipating future needs were also considered valuable.

### Topic 2: Alignment of current rehabilitation services with the normative needs of individuals with traumatic injuries

*Normative needs*. Health professionals expressed frustration at their inability to provide legally mandated services. This often necessitated assuming roles outside their job descriptions to advance the rehabilitation process. Financial difficulties among patients were frequently cited as a barrier to rehabilitation progress. Participants from both specialist and community services advocated for more social workers to assist with financial issues and navigation of social services.

A prevailing theme was the lack of community-based services for patients with cognitive and emotional problems. Participants reported that patients were often denied access to public mental health services due to not meeting admission criteria or the complexity of their issues, leading to worsening conditions over time. Some participants noted that the mental health service was not equipped to manage patients with severe mental health problems in addition to complex injury-related disabilities. Cognitive functioning was identified as a critical determinant of rehabilitation outcomes, yet also as a major challenge. While patients with cognitive impairments often received adequate support in structured, specialized in-patient rehabilitation settings, they faced increased difficulties upon discharge into community settings. These challenges were exacerbated by reduced daily structure, increased demands, and insufficient understanding and accommodation of their impairments. Participants highlighted the lack of knowledge concerning cognitive impairments among community-based rehabilitation professionals, resulting in inadequate management.

Rehabilitation professionals noted that patients often expressed needs beyond practical support and basic care. They reflected on whether rehabilitation services addressed the patients’ psychosocial needs and measures to improve quality of life and meaningful participation. They also described the challenges in prioritizing rehabilitation needs against available resources.

### Topic 3: Reasons why provided rehabilitation services might not be consistent with the needs of individuals with traumatic injuries

*Lack of resources*. A lack of financial resources was among the most frequently reported reasons why services were not in line with normative rehabilitation needs. Participants also stated that restrictions in how long they were able to provide rehabilitation services hampered progress.

*Knowledge/skills*. Participants noted a lack of professional competency standards for community rehabilitation professionals beyond formal education. This deficiency in experience and knowledge regarding issues relating to traumatic injuries hindered the identification of needs and provision of appropriate services. Some health professionals expressed frustration over being assigned tasks without proper training.

*Service organization*. Participants found it challenging to identify available community rehabilitation services. Furthermore, caseworkers responsible for service allocation had varying levels of expertise, leading to inconsistent evaluations of patient needs. Significant geographic variations in service offerings were reported, not only between urban and rural areas but also among different districts within urban areas.

### Topic 4: Perspectives on how the gap between met and unmet rehabilitation needs can be addressed

*Identifying rehabilitation needs*. To identify patients who may need rehabilitation, the participants expressed that patients should receive a comprehensive assessment as early as possible after the injury. They also suggested that some patients would benefit from longer-term rehabilitation in the specialist healthcare setting, while also pointing to a need for more multidisciplinary rehabilitation teams in the community. The transition from hospital to the community was seen as an especially sensitive stage, and participants stated that having more short-term transitional rehabilitation places allowing further assessment of functioning and ability to live independently would be highly beneficial. The importance of having well-functioning communication and collaboration platforms between the specialist and community-based rehabilitation services was conveyed. The health professionals called for changes in organizational structure that would ease interaction between different healthcare levels.

*Perception of rehabilitation*. The rehabilitation professionals advocated for a shared understanding of what community-based rehabilitation should entail. They felt that rehabilitation in the community too often was understood as merely restoration of physical functions and practical assistance, while the broader focus on quality of life and well-being was lost. Participants suggested increased focus on rehabilitation in programmes educating future healthcare professionals as one approach to changing these attitudes.

## DISCUSSION

This qualitative study aimed to capture health professionals’ perspectives on rehabilitation needs following physical traumatic injuries, and gaps between needs and provision of healthcare services, as well as potential areas for improvement. The participants represented both specialist and community-based rehabilitation services; nevertheless, gaps between rehabilitation needs and service provision and suggestions for improvement were mainly identified in community-based services.

The participants reflected on normative standards for high-quality rehabilitation services and highlighted the value of transdisciplinary collaboration. Multidisciplinary rehabilitation is a team effort between different professionals with the overarching goal of improving functioning, treatment, and patient care. While this is generally considered key to high-quality care (22), rehabilitation service provision may be fragmented in the municipalities and multidisciplinary expert competency is sometimes limited. Structured rehabilitation plans established in the specialized care setting may be an important resource for these professionals, but the results indicate a need to strengthen multidisciplinary collaboration in the communities.

Continuity of care as a prerequisite for optimal rehabilitation services following trauma was another important normative standard described in this study. Care continuity, a multidimensional construct involving delivery of services in a logical, coherent, and timely manner, may be gained through trusting relationships and reciprocal understanding developed over time with a team who know the patients well (23). Sustaining a traumatic injury can result in a state of vulnerability (24) and ensuring a predictable rehabilitation process by promoting in patients a sense of trust and safety is essential. Receiving continuous information and having one’s needs anticipated is associated with interpersonal trust, and feelings of being important, valued, and assurance in patients (25). Accurate and timely information-sharing and well-executed transitions between healthcare levels have several benefits such as improved care coordination and quality of care, patient safety while reducing medical errors, and healthcare costs (26).

The community-based health professionals expressed frustration with not being able to provide necessary services despite this being required by law. Norway is a welfare state with universal access to healthcare services, and all municipalities are mandated to provide a range of services, including a medical doctor, psychologist, physiotherapist, nurse, and occupational therapist. While introducing laws that stipulate what services should be available is an important step towards equal access to rehabilitation, many communities still struggle to ensure availability. This is highlighted in a recent report from the Office of the Auditor General of Norway, finding that more than 85% of Norwegian municipalities lack legally required rehabilitation services (27). The participants also expressed a need for more social workers in the community, a service that is not currently legally mandated. Insufficient community-based rehabilitation services have also been recognized internationally (28). The reported geographic variations in service availability are also in accordance with previous research (5, 29). Notably, participants not only expressed variations between urban and rural areas, but also between different districts in urban areas.

Cognitive dysfunction and mental health problems following traumatic injuries are significantly associated with diminished quality of life in patients and their family members (30, 31). The study participants reported that there was insufficient knowledge regarding cognitive impairment and appropriate management strategies in the primary healthcare service. Additionally, the public mental health service was not equipped to manage the complexity of trauma-related consequences. Studies on health professionals working with other patient populations with cognitive impairment have documented poor recognition of cognitive problems and limited knowledge and management skills (32, 33). Lack of training and confidence in managing cognitive and mental health problems in trauma patients, in addition to resource restrictions, may contribute to these problems going undetected and under-treated.

The rehabilitation professionals felt a need to focus on practical support and basic care due to limited resources, while the patients themselves expressed a wish for rehabilitation measures focused on how to live a meaningful life with injury-related impairment. This leads to reflections around person-centred care and rehabilitation goal setting that should be meaningful and based on fundamental beliefs, goals, and attitudes of the patient (34). Participants further described that they could not provide rehabilitation services long enough and felt that this could hamper recovery. Recovery can be viewed as a process towards resuming a meaningful life and long-term services typically provided in the community need to include comprehensive support in the long term including integrated, person-centred care (35).

Participants advocated for a shift in how community-based rehabilitation is perceived. According to the WHO, community-based rehabilitation should involve attempts to enhance the quality of life of persons with disabilities and their families, going beyond basic needs to promote participation and inclusion in several areas including health, work, socialization, and a sense of empowerment (36). A recent Norwegian publication reported that the allocation of community-based rehabilitation services mainly focused on medically oriented care, without considering psychosocial needs or measures to increase activity and participation (37). The participants reflected on how increased focus on rehabilitation in the programmes educating future rehabilitation professionals may be a way of broadening the perspective. This is in accordance with findings from a review investigating the training needs of community-based rehabilitation workers that highlighted the lack of standardized training and the need for further training in clinical, social, management, and cultural competence skills (38).

The health professionals reported a need to establish collaborative arenas between specialist and community-based rehabilitation services. Previous studies have underlined the importance of organizational structures that stimulate communication within and between different levels of healthcare (39, 40). In Norway, specialized care and community-based services are both responsible for the coordination of rehabilitation services and several measures to improve care coordination and collaboration have been introduced, such as hospital- and community-based care coordinators. However, unclear responsibility for information sharing across organizational boundaries, not having access to written information, and variations in rehabilitation culture have been identified as communication barriers in rehabilitation (40). Moreover, although there are considerable organizational differences even across the European countries, community-based services represent the main platform for rehabilitation services in the post-acute and chronic phase. The study findings reflect internationally recognized gaps and recommendations for improving rehabilitation. The WHO’s Rehabilitation 2030 initiative, NICE guidelines, and the European Society for Trauma and Emergency Surgery (ESTES) recommendations all emphasize system-level planning, multidisciplinary coordination, and continuity of care. However, persistent barriers such as weak organization, poor coordination, limited resources, and variable workforce competencies continue to impede high-quality trauma rehabilitation, underscoring the need for stronger policy commitment and practical implementation.

This study used a qualitative approach informed by an evidence-based needs framework and involvement of rehabilitation professionals to identify gaps between normative needs and provision of rehabilitation services to individuals with traumatic injuries. Participants represented both specialist and community-based rehabilitation settings from urban and rural geographic areas. However, they did not represent all occupational backgrounds, and females employed in the specialist healthcare setting represented two-thirds of the participants. The researchers who performed this study are rehabilitation professionals and the identified themes should be viewed taking this into consideration. We acknowledge that our professional background may have influenced interpretation of the data material and identified themes, with the risk of certain themes unintentionally being overlooked or emphasized. On the other hand, our thorough understanding of rehabilitation may have contributed to an understanding of nuances in the informant interviews.

In conclusion, this study highlights the need to strengthen community-based rehabilitation services for individuals who sustain traumatic injuries. Efforts to increase knowledge concerning injury-related consequences and the skills to manage these, organizational structures that ease information transfer and cooperation between specialist and community-based services, and promoting an understanding of community-based rehabilitation which includes measures that cover psychosocial needs may be means of improving current rehabilitation service provision. Thus, improving existing services involves critical reflection at different healthcare levels, including patient care, service organization, and healthcare policy.
